# Neural mechanism of conscious perception for potential clinical applications

**DOI:** 10.1002/ctm2.70387

**Published:** 2025-09-19

**Authors:** Yong Wang, Shouyang Yu, Yueqing Dong, Yuanyuan Dang, Hulin Zhao, Mingsha Zhang, Xiaoli Li

**Affiliations:** ^1^ Department of Rehabilitation Medicine Zhujiang Hospital Southern Medical University Guangzhou China; ^2^ School of Anesthesiology Key Laboratory of Anesthesia and Organ Protection of Ministry of Education (In Cultivation) Zunyi Medical University Zunyi China; ^3^ Xincheng Hospital of Tianjin University Tianjin China; ^4^ Department of Neurosurgery Chinese PLA General Hospital Beijing China; ^5^ State Key Laboratory of Cognitive Neuroscience and Learning and IDG/McGovern Institute for Brain Research Division of Psychology Beijing Normal University Beijing China; ^6^ PAZHOU LAB Guangzhou China

The generation of consciousness has remained one of the most enduring scientific quests in human history. When a visual stimulus enters the eye, how does the brain transform it into the subjective experience of “I see”? Traditional theories posited the cerebral cortex as the epicenter of conscious perception, relegating the thalamus to a mere relay station for sensory signals. However, a groundbreaking study recently published in Science has overturned this paradigm, identifying the mediodorsal thalamic nuclei (MDm) and intralaminar nuclei (ILN) as the true “gatekeepers” of conscious perception.^1^ Through dynamic connectivity with the prefrontal cortex, these nuclei determine which sensory information gains access to the conscious perception. What groundbreaking clinical implications might this discovery yield?

## 1 THE CONCEPT OF CONSCIOUS PERCEPTION

Conscious perception, is one of the most complex issues in the field of cognitive science, meaning subjects convert external stimuli into subjective experiences through neural activity. Conscious perception involves the awareness and understanding of one's environment, internal stimuli, and cognitive processes. As a key capability for human cognition, it enables individuals to be aware of events, emotions, thoughts, and bodily states. From the perspective of neuroscience, conscious perception is a process that involves several brain regions, particularly the thalamus and the prefrontal cortex.^2^, ^3^ These regions synthesize information from sensory systems, such as vision, hearing, and touch, to construct a clear conscious experience.

## 2 CONSCIOUS PERCEPTION IMPAIRMENTS ACROSS NEUROLOGICAL AND PSYCHIATRIC DISORDERS

**FIGURE 1:**
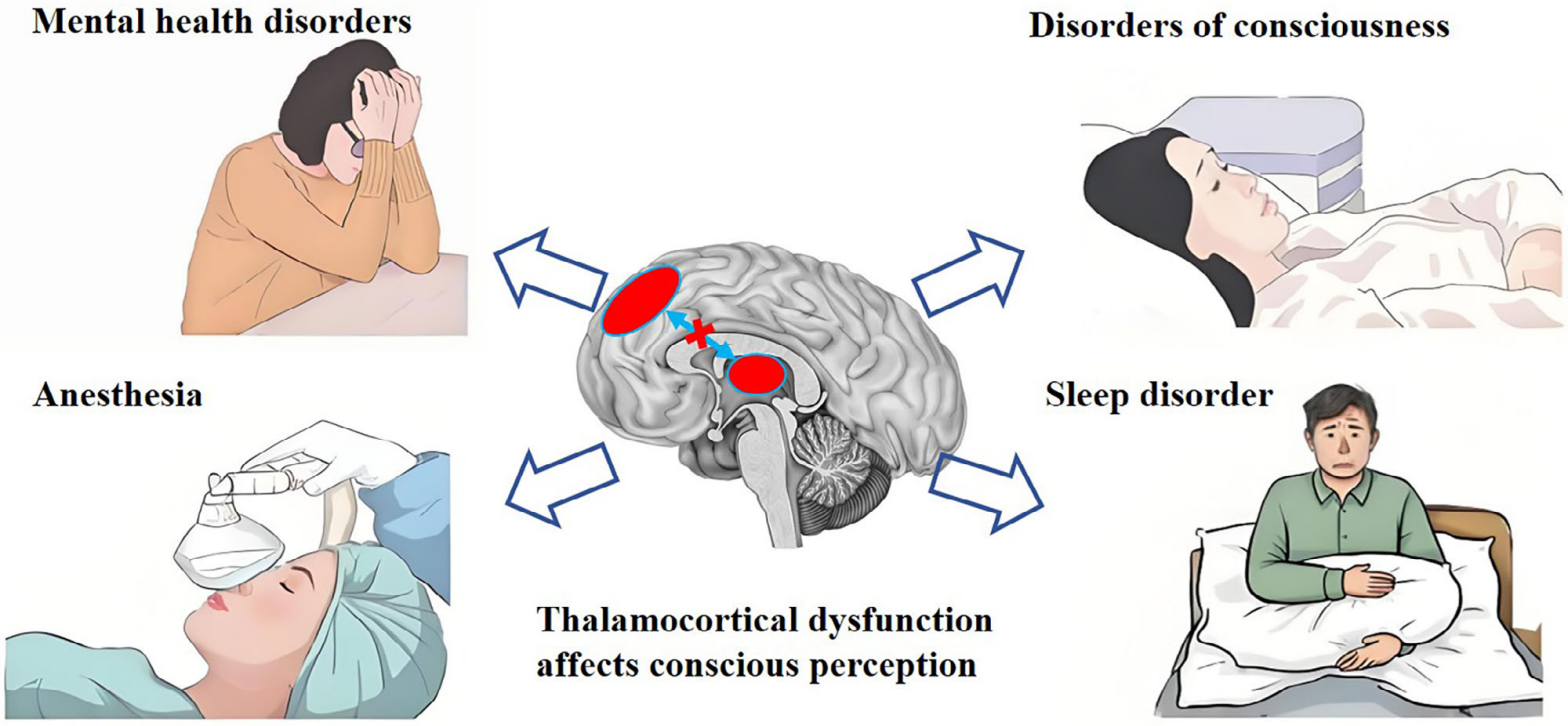
Impairments of conscious perception in neurological and psychiatric disorders. Perceptual awareness arises from the integration of thalamocortical activity. Disruptions in thalamocortical function can lead to impairments in conscious perception, contributing to disorders of consciousness, sleep disturbances, effects of anesthesia, and various psychiatric conditions.

Conscious perception is impaired in various medical conditions (see Figure 1). Neurodegenerative diseases, including Alzheimer's and Parkinson's, are linked to progressive deterioration in awareness and perception. Individuals with Alzheimer's disease often exhibit a decline in consciousness and self‐awareness,^4^ whereas those with Parkinson's may endure visual perception problems due to the loss of dopamine‐producing cells in the retina.^5^ Other conditions that affect conscious perception include epilepsy, which can cause sensory disturbances; and sleep disorders, which can lead to perceptual changes due to sleep deprivation or disrupted circadian rhythms (Figure 1).

### 2.1 Disorders of consciousness

Severe brain injury leads to disorders of consciousness (DoC), and etiology includes cardiac arrest, traumatic brain injury, haemorrhage and stroke. DoC is characterized by alterations in arousal and/or awareness, manifesting as loss of self/environmental awareness and impaired external communication.^6^ DOC are categorized as vegetative state/unresponsive wakefulness syndrome (VS/UWS) or minimally conscious state (MCS) based on consciousness‐related behavioral activities. The mechanism is a widespread dysfunction of the corticothalamic system that leads to broad withdrawal of excitatory synaptic activity across the cerebral cortex, producing inhibition of components of the central thalamus. The severity levels of thalamocortical deafferentation reflect consciousness level, thalamic lesions are notably less prevalent in patients diagnosed with MCS compared to VS/UWS. MCS patients preserve the relative long‐range corticothalamic connections and frontoparietal network and retain the capacity for cognitive processing and external sensory awareness. Functional connectivity is a diagnostic biomarker of consciousness and a potential predictor of ongoing recovery. The effective connectivity from the central thalamic CM/Pf nucleus to the anterior medial prefrontal cortex (aMPFC) (ECCM/Pf aMPFC) is a key indicator for distinguishing functional recovery in patients, complementing traditional behavioral scales like the Coma Recovery Scale‐Revised (CRS‐R).^7^

The mesocircuit thalamocortical model explains the potential mechanisms of action of various therapeutic interventions Deep brain stimulation (DBS) targeting the central thalamus could improve therapeutic precision.^8^ Direct stimulation of the CL using DBS and low‐intensity focused ultrasound pulsation techniques stimulates the thalamocortical connectivity that supports the functional activation of the frontal cortex. Transcranial direct current stimulation and transcranial magnetic stimulation targeting the prefrontal cortex increased fronto‐parietal connectivity and induced a stronger connectivity between the prefrontal cortex and the thalamus facilitating recovery from consciousness.^9^

### 2.2 Sleep disorder

During sleep, the reticular thalamic nucleus (RTN), acts as another gatekeeper controlling sensory information flowing from the thalamus to the cortex through inhibition of the thalamus.^10^ During NREM sleep, consciousness gradually declines as the brain transitions through stages N1 to N3, accompanied by a reduction in the amount of external stimuli reaching the cortex. In contrast, REM sleep is typically associated with vivid conscious experiences in dreams despite disconnection from the external environment. Sleep disorders affect a significant portion of the population, impacting their quality of life and overall health. Recent studies suggest that alterations in nocturnal consciousness may be related to the occurrence of sleep disorders, such as insomnia and NREM arousal parasomnias.

Insomnia is the most common type of sleep disorder. During sleep, the inhibition of higher‐order thalamic areas by the RTN reduces the sensory information flowing from the thalamus to the cortex, which is thought to facilitate sleep onset and sleep maintenance. On the contrary, if the reticular thalamic nucleus (RTN) fails to effectively inhibit the sensory information flowing from the thalamus to the cortex, the brain may retain a certain level of awareness, which can lead to difficulty in falling asleep, frequent awakenings, or early morning awakenings, thus resulting in insomnia. These pieces of evidence suggest that enhancing the inhibition of higher‐order thalamic areas by the RTN may play a therapeutic role in treating insomnia.^11^ NREM arousal parasomnias, including confusional arousals, sleepwalking, and sleep terrors, are paroxysmal abnormal nocturnal behaviors, usually arising from stage 3 or 4 NREM sleep.^12^ These behaviors emerge when the cortex incompletely arouses from deep NREM sleep due to external or internal stimuli. The mechanism may be related to the incomplete inhibition of higher‐order thalamic areas by the reticular thalamic nucleus (RTN). Reducing the inhibitory effect of the RTN on higher‐order thalamic areas may play a therapeutic role in treating NREM arousal parasomnias.

### 2.3 Anesthesia

The process of consciousness loss induced by anesthesia involves distinct neural activity changes in the cortex and thalamus.^13^ Anesthetic agents such as propofol and isoflurane significantly reduce thalamic neuronal firing frequency by the inhibitory neurotransmitter GABA. This prevents external sensory signals (e.g., tactile, auditory) from reaching the cortex, resulting in loss of perception of external stimuli. Cortical neurons exhibit decreased firing rates alongside widespread synchronized slow waves (delta waves, 0.5‐4 Hz), resembling the electroencephalographic (EEG) patterns of deep sleep. These synchronized slow waves disrupt information exchange between cortical regions, leading to the disintegration of higher‐order cognitive functions (e.g., decision‐making, self‐awareness).^14^ The bidirectional thalamocortical connections, such as the thalamus prefrontal cortex loop, are blocked by anesthetics, preventing cross‐regional information integration. The consciousness loss may lie in the thalamus “shutting down” external sensory input while the cortex loses its capacity to synthesize information into unified experiences.

Postoperative Delirium (POD) is an acute cerebral dysfunction characterized by fluctuating consciousness levels, attentional deficits, cognitive disturbances, and perceptual abnormalities (e.g., hallucinations or illusions) within the early postoperative period (typically 24–72 h).^15^ Anesthetics like propofol may exacerbate postoperative cerebral dysfunction through GABAergic overinhibition; excessive anesthetic depth inducing EEG burst‐suppression patterns correlates with delirium risk; anesthesia and postoperative environments disrupt melatonin secretion, impairing circadian rhythms and cognitive integration. Elderly patients, due to brain atrophy, neuronal loss, and reduced cerebrovascular reactivity, are more susceptible to delirium triggered by hypoxia, hypoperfusion, or metabolic disturbances. Their neurodegenerative vulnerability and metabolic frailty make them high‐risk populations. Neuromodulation techniques offer novel therapeutic approaches by regulating brain network activity, suppressing neuroinflammation, or enhancing neurotransmitter function. POD is closely associated with functional decoupling in thalamocortical circuits, while non‐invasive interventions like transcranial magnetic stimulation (TMS) or transcranial direct current stimulation (tDCS) may restore cortical excitability and network synchronization.^16^ For instance, low‐frequency TMS could suppress hyperactive cortical regions, whereas high‐frequency stimulation might improve cerebral hypoactivity in hypoactive delirium subtypes.

### 2.4 Mental health disorders

Mental health disorders also significantly impact conscious perception. Schizophrenia is characterized by perceptual abnormalities that lead to hallucinations and delusions, often due to disrupted neurotransmitter signaling and cortical plasticity. Treatment‐resistant depression (TRD) is characterized by perceptual‐cognitive dysfunction, manifesting as depressed mood, psychomotor retardation, and reduced volition. Autism spectrum disorder (ASD) involves sensory symptoms linked to altered neural circuits and impaired GABAergic signaling, resulting in sensory hypersensitivity or hyposensitivity. Attention deficit hyperactivity disorder (ADHD) patients often struggle with sensory overload due to impaired information processing and perceptual deficits. Sensory gating dysfunction in sleep leads to sleep disorders such as insomnia and NREM arousal parasomnias, which are related to abnormal inhibition of the cortex by the reticular thalamic nucleus (RTN).^11^, ^12^

In psychiatric disorders, the thalamofrontal “gating” mechanism offers mechanistic explanations for perceptual anomalies.^1^ The disrupted sensory integration in schizophrenia may arise from impaired CM‐LPFC θ‐phase coupling, indicating therapeutic potential in theta‐burst stimulation (TBS) protocols to restore frontothalamic coherence.^17^ For treatment‐resistant depression, a combination of deep brain stimulation (DBS) targeting the MDm and ventral striatum could synergistically modulate affective‐cognitive networks, building on existing ventral capsule/ventral striatum (VC/VS) DBS paradigms.^18^

## 3 SUMMARY

This paradigm shift, from cortical‐centric to thalamocortical network models of consciousness, calls for multidisciplinary collaboration. By integrating sEEG‐guided neuromodulation, computational modeling of mesocircuit dynamics, and machine learning‐based biomarker discovery, clinicians can develop precision therapies that bridge molecular mechanisms with systems‐level network reorganization. These advances not only redefine our approach to DoC but also offer novel pathways for treating perceptual‐cognitive deficits across neurological and psychiatric disorders.
